# Analysis of Antidepressant Activity of Huang-Lian Jie-Du Decoction Through Network Pharmacology and Metabolomics

**DOI:** 10.3389/fphar.2021.619288

**Published:** 2021-03-04

**Authors:** Shu-Yue Qu, Xiao-Yue Li, Xia Heng, Yi-Yu Qi, Ping-Yuan Ge, Sai-jia Ni, Zeng-Ying Yao, Rui Guo, Nian-Yun Yang, Yi Cao, Qi-Chun Zhang, Hua-Xu Zhu

**Affiliations:** ^1^ Jiangsu Key Laboratory for High Technology Research of TCM Formulae, Nanjing University of Chinese Medicine, Nanjing, China; ^2^ Jiangsu Key Laboratory for Pharmacology and Safety Evaluation of Chinese Materia Medica, School of Pharmacy, Nanjing University of Chinese Medicine, Nanjing, China; ^3^ School of Medicine and Holistic Integrative Medicine, Nanjing University of Chinese Medicine, Nanjing, China; ^4^ Institute of Literature in Chinese Medicine, Nanjing University of Chinese Medicine, Nanjing, China

**Keywords:** network pharmacology, huang-lian jie-du decoction, depression, tryptophan metabolism, metabolomics

## Abstract

Depressive disorder is a common mental disorder characterized by depressed mood and loss of interest or pleasure. As the Herbal medicines are mainly used as complementary and alternative therapy for depression. This study aimed at exploring antidepressant activity of Huang-lian Jie-du Decoction (HLJDD), and evaluating active components and potential depression-associated targets. HLJDD was administered on chronic unpredictable mild stress-induced (CUMS) depressive mice. Behavior evaluation was performed through force swimming test (FST), novelty-suppressed feeding test (NSF), and open field test (OFT). Active components of HLJDD, potential targets, and metabolic pathways involved in depression were explored through systemic biology-based network pharmacology assay, molecular docking and metabonomics. FST assay showed that CUMS mice administered with HLJDD had significantly shorter immobility time compared with control mice. Further, HLJDD alleviated feeding latency of CUMS mice in NSFand increased moving distance and duration in OFT. In the following network pharmacology assay, thirty-eight active compounds in HLJDD were identified based on drug-like characteristics, and pharmacokinetics and pharmacodynamics profiles. Moreover, forty-eight molecular targets and ten biochemical pathways were uncovered through molecular docking and metabonomics. GRIN2B, DRD, PRKCA, HTR, MAOA, SLC6A4, GRIN2A, and CACNA1A are implicated in inhibition of depressive symptoms through modulating tryptophan metabolism, serotonergic and dopaminergic synaptic activities, cAMP signaling pathway, and calcium signaling pathway. Further network pharmacology-based analysis showed a correlation between HLJDD and tryptophan metabolism. A total of thirty-seven active compounds, seventy-six targets, and sixteen biochemical pathways were involved in tryptophan metabolism. These findings show that HLJDD acts on potential targets such as SLC6A4, HTR, INS, MAO, CAT, and FoxO, PI3K/Akt, calcium, HIF-1, and mTOR signaling pathways, and modulates serotoninergic and dopaminergic synaptic functions. In addition, metabonomics showed that tryptophan metabolism is the primary target for HLJDD in CUMS mice. The findings of the study show that HLJDD exhibited antidepressant effects. SLC6A4 and MAOA in tryptophan metabolism were modulated by berberine, baicalein, tetrahydroberberine, candicine and may be the main antidepressant targets for HLJDD.


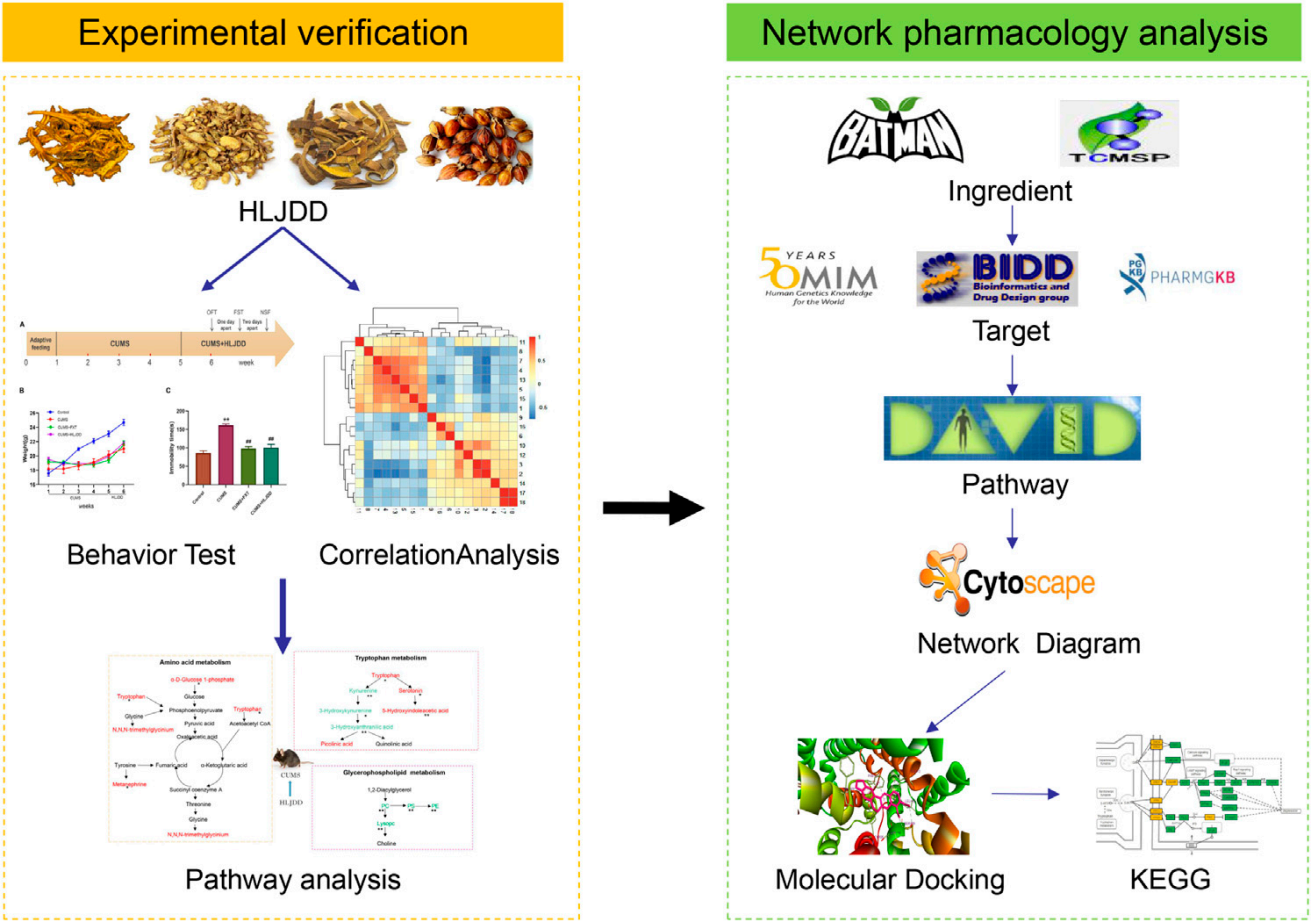


## Highlights

1. HLJDD ameliorates the depression-like behaviors in CUMS-induced depressive mice.
2. Tryptophan metabolic plays a key role in underpinning the antidepressant effect of HLJDD.
3. SLC6A4 and MAOA are the most potential targets for HLJDD on depression.

## Introduction

Major depressive disorder (MDD) is a common and severe mental disorder. MDD patients present with depressed mood, pessimistic thinking, loss of enjoyment in usual activities and lack of energy. Worldwide, more than 350 million people are suffering from depression (Ge et al., 2020; Wigner et al., 2018). World Health Organization reports that depression is a major cause of disabilities in the world. Depression reduces quality of life, increases risk of death at any age, and is a health burden on families and society (Guo et al., 2017).

Hypertension patients treated with reserpine often present with depression (Freis, 1954), therefore, Joseph Schildkraut proposed the monoamine hypothesis of depression in 1965. Compounds targeting 5-HT metabolism have antidepressant activity. Newly synthesized 5-HT from tryptophan is transported into synaptic vesicles within presynaptic neurons. Upon stimulation, 5-HT is released from vesicles into the synaptic space where it interacts with several postsynaptic and presynaptic serotonin receptors. Transportation of 5-HT back into presynaptic neurons by SLC6A4 or breakdown of 5-HT into 5-hydroxyindoleacetic acid by MAOA terminates activity of 5-HT.

Although more than a dozen antidepressants are available, most individuals with depression do not respond to these treatments. Most antidepressants are associated with unfavorable long-term outcomes and paradoxical effects, such as induction of depression, worsening of symptoms and withdrawal symptoms (Chouinard and Chouinard, 2015; Fava, 2003). Multi-target chemicals such as paroxetine and vortioxetine have been developed to reduce side effects, intolerance and improve efficacy of antidepressant drugs (Bang-Andersen et al., 2011; Cipriani et al., 2018). Use of natural products that contain diverse chemical components and acceptable tolerance following long-term administration can be used as alternative therapy against depression.

In addition to use of complementary and alternative medicine as prophylactic interventions or treatments as, there is increased interest in use of Chinese herbal medicine for depression treatment. Huang-lian Jie-du decoction (HLJDD), is a well-known Chinese herbal initially recorded in Prescriptions for Emergent Reference over 1500 years ago (Zhouhou Beiji Fang, A.D. 341). HLJDD has heat dissipation and detoxification activities (Dai et al., 2018). It is a mixture of Coptis chinensis Franch, Scutellaria baicalensis Georgi, Phellodendron amurense Ruer and Gardenia jasminoides J. Ellis herbs with a weight ratio of 3:2:2:3. HLJDD is used for treatment of symptoms caused by heat toxins (Yue et al., 2014). Several studies report that HLJDD ameliorates multiple central nervous system diseases such as cerebral ischemia, Alzheimer’s disease (AD) and mental disorder (Liu et al., 2019; Okamoto et al., 2013; Wang et al., 2013). The formula modulates monoamine metabolism and kynureninet metabolism which are both implicated in pathogenesis of depressive syndromes (Sasaki et al., 2000; Yu et al., 2015). Active components of the formulation such as berberine and baicalin exhibit antidepressant activities (Lee et al., 2012; Zhang et al., 2016). HLJDD is believed to improve depression through synergistic activities of the active components. However, specific relationships between depression and HLJDD, and potential mechanisms of action of specific components have not been fully elucidated.

Network pharmacology is an emerging approach for evaluating systematic mechanism of action for multi-target drugs based on biological network associating with a disease. The approach is used to explore biological basis of herbal formulae and bioactive ingredients present. Several herbal formulae for treatment of neurological or mental disorders have been developed through network pharmacology (Zhang et al., 2019). HLJDD modulates ischemic stroke through “shotgun-like” pharmacological mechanism (Wang et al., 2019). Tian-Ma-Gou-Teng-Yin ameliorates Alzheimer’s disease through neuroprotective and anti-neuroinflammatory activities (Wang et al., 2018). Key active compounds and antidepressant mechanism Xiao-Yao-San in treatment of depression were explored through network pharmacology (Liu et al., 2020).

In this study, experience-based approach and evidence-based assay were used to explore antidepressant activities of HLJDD, a traditional Chinese medicine formula. We explored the symptoms for depression in CUMS-induced depressive animal model after treatment with HLJDD. Further, network pharmacology assay using bioinformatics tools, systems biology, and polypharmacology was performed to determine genes, proteins, and signaling pathways implicated in activity of HLJDD against depression (Zhang et al., 2019). Currently, application of network pharmacology successfully integrates systematic information on herbal medicines or formulas of TCM for various diseases (Zhang et al., 2019; Ge et al., 2020). Findings of this study indicate that HLJDD ameliorates depressive-like behaviors in CUMS mice. In addition, the formulation modulates serotonergic synapse function and tryptophan metabolism. Further, we determined interactions between candidate chemicals and their molecular targets and analyzed their binding energy scores. Finally, we carried out metabonomics analysis to verify underlying mechanisms of action for HLJDD against depression in mice. Findings of the study show that tryptophan metabolism is the main target for HLJDD. This information provides a basis to design and develop novel plant-based therapies against depression.

## Animal Experiments

### Chemicals and Materials

Rhizome Coptis (Coptis chinensis Franch., Ranunculaceae; batch number, 1711028050), RadixScutellariae (Scutellaria baicalensis Georgi, Lemnaceae; batch number, 1709019024), CortexPhellodendri amurensis (Phellodendron amurense Ruer., Rutaceae; batch number, 1709016005) and Fructus Gardeniae (Gardenia jasminoides Ellis., Rutaceae; batch number, 1711018022) were purchased from Fu Chun Tong Chinese Herbal Pieces Co., Ltd. (Anhui, China). Herbal materials were authenticated by Dr Qinan Wu (Professor of Pharmacognosy, College of Pharmacy, Nanjing University of Chinese Medicine, Nanjing, China). A voucher number was assigned to each specimen and voucher specimens were deposited in Nanjing University of Chinese Medicine. Fluoxetine HCL (FXT) was purchased from Beijing Yinuokai Technology Co., Ltd. (Beijing, China).

### Preparation of Huang-Lian Jie-Du Decoction Extract

HLJDD extract was prepared according to a protocol described in our previous study (Zeng et al., 2010). Coptis chinensis Franch (270 g), Scutellaria baicalensis Georgi (180 g), Phellodendron amurense Ruer (180 g) and Phellodendron amurense Ruer (270 g) mixture was soaked in 9 L of distilled water for 1 h and then refluxed for 1.5 h. Dregs of the herbs were refluxed again in 7.2 L of water for 1 h. The two supernatants were mixed and filtered using gauze. The supernatant was concentrated to 1 L using a rotary evaporator. Further, the supernatant was frozen at −80°C for 12 h. The frozen was lyophilized using a freeze dryer resulting to 222 g of lyophilized powder. Analysis of the constituents of HLJDD was carried out using HPLC with berberine, baicalin and geniposide as standards (Agilent 1260, United States). Peak areas of the samples were substituted into a standard curve, and the abundances of berberine, baicalin, and geniposide in HLJDD were 7.67%, 11.46%, 2.42% respectively. HLJDD powder used for administration to mice was suspended in 0.50% carboxymethyl cellulose sodium salt (CMC-Na) aqueous solution.

### Animals

SPF male C57BL/6 mice, weighing 18–22 g (Animal Multiplication Center of Qigong Mountain, Nanjing, China), were housed in a animal laboratory with regulated temperature and humidity of 25 ± 1°C and 60 ± 5%, respectively. Animal were acclimatized for a week and randomly divided into 4 groups: control group, model group (CUMS), fluoxetine hydrochloride group (CUMS + FXT, 20 mg/kg) and HLJDD group (CUMS + HLJDD, 590 mg/kg). HLJDD and FXT were dissolved in 0.5% CMC-Na. A total of 20 ml/kg body weight for both HLJDD and FXT was administered orally every week. Mice in the control and model group were given 0.5% CMC-Na. HLJDD dosage was calculated using dosage conversion relationship between mice and human and clinical dosage of HLJDD extract. Behavioral experiment was performed 1 h after the last administration and data were analyzed using Video Analysis System for Animal Behavior (JL, Shanghai, PRC). The study was carried out following recommendations provided by Guide for the Care and Use of Laboratory Animals of the National Institutes of Health (NIH Publication No. 80–23; revised 1978). The Animal Care and Use Committee of Nanjing University of Chinese Medicine approved the protocol and total number of mice used in this study.

### Chronic Unpredictable Mild Stress Model of Depression

Chronic unpredictable mild stress (CUMS) mice model is developed by exposing animals to a set of unpredictable mild stressors for several weeks to induce depression-like phenotypes (Wu et al., 2012). In this study, mice were subjected to CUMS for 4 weeks using food deprivation (24 h), water deprivation (24 h), cage tilting at 45° without bedding (24 h), dampening sawdust with 125 ml of clean water (24 h), removing sawdust from the cage (24 h), subjecting animals to cold swim (4°C, 5 min), hot environment (45°C, 5 min), tail clip (2 min, 1 cm from the distal portion of the tail), physical restraint (2 h) and shaking the cage (40 min) as stressors. Each animal was exposed to two of the ten mentioned stressors each day. Induction of depression using different stressors was randomized. After subjection to stressors for each day, mice were placed in clean cages and returned to the housing facility.

### Forced Swim Test

Mice exhibits behavioral despair under unavoidable stressors, which is similar to MDD phenotypes in human (Steru et al., 1985). Forced swim test (FST) is used for determination of efficacy of antidepressant treatments (Rubio et al., 2006). CUMS mice models were housed in the experimental room 2 h before the test for acclimatization. Oral administrations of the treatment and control were carried out 60 min before starting FST. FST was carried out by placing each animal at a depth of 10 cm in an open cylindrical container (12 cm diameter ×25 cm height) containing water (25 ± 1°C). Mice were not allowed to touch the bottom of the cylinder. Each animal was allowed a 6-min swim test session. Swimming behavior of each animal was recorded, and the last 4 min of the session analyzed.

### Novelty-Suppressed Feeding Test

Novelty-suppressed feeding test (NSF) is an effective behavioral paradigm commonly used to evaluate depression-like behaviors in experimental animals after fasting. The test uses the conflict between feeding and fear of a novel environment (Dulawa and Hen, 2005). After subjecting mice to fasting for 24 h, they were acclimatized in the testing room f overnight. A platform with food pellets was placed at the center of a square open box (40 × 40 × 35 cm, length × width × height). Each animal was placed in the test apparatus and the same position and direction to the platform was maintained. Time taken for each animal to eat the food pellet (bite the food pellet with use of forepaws while sitting on its haunches) was recorded during the 5 min run time.

### Open Field Test

Open field test (OFT) is used to measure of exploratory behavior, locomotor activity and general depressive-like activity (Bai et al., 2012). Mice were transported to the experimental room 2 h prior to the test to adapt to the environment. OFT apparatus was constructed using four cubic boxes (40 × 40 × 40 cm, length × width × height) made of blue plastic material leaving the top open. The bottom of each arena was divided into 16 square lattices. Mice were placed at the center of the arena 1 h after administration with HLJDD, FXT or vehicle. Mice were allowed to explore the arena for 5 min. Time taken for central movement, distance moved at the center, total duration and total distance of movement of CUMS mice were recorded and analyzed using automated video tracking to evaluate exploratory behavior and response to novelty.

### Neurotransmitters Assay

Neurotransmitters including *γ*-aminobutyric acid (GABA), glutamic acid (Glu), 5-hydroxytryptamine (5-HT), dopamine (DA) and acetylcholine (Ach) in the hippocampus, cortex, striatum and amygdala were determined using LC-MS/MS. Accurately weighed cerebral tissues were added to 10-fold volume of 0.1% formic acid and completely homogenized for 3 min. 100 μL of the homogenate were added to 200 μL 0.2% formic acid acetonitrile, vortexed for 3 min and centrifuged at 12,000 rpm at 4°C for 10 min. Supernatants were concentrated and reconstituted with 0.1% formic acid.

A 2.1 mm × 100 mm, 2.7 μm chromatographic column (Infinity Lab Poroshell 120 EC-C18，Agilent, United States) was used. Other chromatographic conditions were; mobile phase consisting of A, 0.1% formic acid in water, and B, acetonitrile; column temperature, 30°C; flow rate: 300 μL/min; injection volume, 2 μL; gradient elution conditions, 0–2 min, 98–95% A; 2–3.5 min, 95–40% A; 3.5–4 min, 40–98% A; 4–6.1 min, 98% **A**. Ionization source conditions were capillary voltage, 3.0 kV; sampling cone voltage, 30.0 V; source temperature, 150°C and desolvation temperature, 500°C.

### Statistical Analyses

Data were presented as mean ± SD. Analysis of variance (ANOVA with a Fisher’s LSD test) was carried out to determine differences between groups. *p* < 0.05 was considered statistically significant. Data analysis was carried out using SPSS 17.0 software.

## Network Pharmacology Analysis

### Databases and Software

TCMSP database (http://lsp.nwu.edu.cn/tcmsp.php), BATMAN-TCM database (http://bionet.ncpsb.org/batman-tcm/), PharmMapper database (http://lilab-ecust.cn/pharmmapper/index.html), OMIM database (http://www.omim.org), PharmGkb database (https://www.pharmgkb.org/), TTD (http://bidd.nus.edu.sg/BIDD- Databases/TTD/TTD.asp), System Dock database Site database (http://systemsdock.unit.oist.jp), String Version 10.5 server (https://string-db.org/), RCSB PDB database (http://www.rcsb.org/pdb/home/home. do), DAVID database (https://david.ncifcrf.gov/) and Pubchem database (https://pubchem.ncbi.nlm.nih. gov) were used for network pharmacology analysis. Cytoscape 3.8.0 software was used to construct an interaction network. AutoDock Vina software was used to predict the binding mode and binding energy scores between active compounds and molecular targets.

### Screening of Chemical Ingredients

Active compounds were screened using TCMSP and BATMAN-TCM databases. TCMSP was used to predict ADME/T and other biological, chemical and physical parameters of active compounds using mathematical and computational models. SysDT model is used to find relevant targets, map diseases, tissues and organs, and to predict drug efficacy and toxicity mechanism (Ru et al., 2014). BATMAN-TCM database is an online tool for study of molecular mechanisms of traditional Chinese medicine. BATMAN-TCM compares submitted herbs to a list of reference ingredients saved in the database, to predict targets for the new ingredients (Liu et al., 2016). In this study, components of HLJDD Huangshan (Coptis chinensis Franch), Huangqin (Scutellaria baicalensis Georgi Scutellaria baicalensis Georgi), Huanghai (Phellodendron amurense Ruer), Zhizi (Gardenia jasminoides J. Ellis) were submitted to BATMAN-TCM and TCMSP database. Active compounds with a Score ≥20 and *p*-value ≤ 0.05 in BATMAN-TCM were selected. Oral bioavailability (OB) ≥ 30% and drug-likeness (DL) ≥ 0.18 parameters in TCMSP were used to screen BATMAN-TCM results. The final cluster of chemical ingredients of HLJDD was a combination of BATMAN-TCM and TCMSP results.

### Screening Ingredient-Related Targets

Targets of the active compounds were predicted using BATMAN-TCM, TCMSP and PharmMapper databases. PharmMapper Server is an open-source web-server for identification of potential target for small molecules (drugs, natural products, or newly discovered compounds with unknown binding targets) using pharmacophore mapping approach (Liu et al., 2010). 3D structural SDF format (.sdf) of these candidate active ingredients of HLJDD were downloaded from PubChem database and submitted to PharmMapper for identification of potential targets.

### Screening Disease-Associated Targets

OMIM database, TTD database, and PharmGkb database were used for identification of potential targets implicated in depression and tryptophan metabolism. Depressive, antidepressant, depressed, depression for depressive symptoms analysis, and tryptophan, and tryptophan metabolism for tryptophan metabolism assay were used as keywords in the three databases and *Homo sapiens* targets were selected for study.

### Gene Ontology and Kyoto Encyclopedia Genes Genomes Enrichment Analysis

Gene Ontology (GO) and Kyoto Encyclopedia of Genes and Genomes (KEGG) were used to analyze signaling pathways and bioprocesses associated with depression that are modulated by HLJDD. Moreover, targets implicated in HLJDD activity and tryptophan metabolism were selected for enrichment assay. Enrichment assay was used to predict possible association between HLJDD and tryptophan metabolism. Further, predicted targets were submitted to DAVID database. OFFICIAL_GENE_SYMBOL item gene list were selected for analysis. GO enrichment analysis was performed together with KEGG pathways analysis.

### Network Diagram Construction

Data files and attribute files of compound-target interaction, target-pathway of depressive disorders and tryptophan metabolism were saved in Microsoft Excel. Data files were subsequently imported to Cytoscape 3.2.1 software and ingredient-target interaction networks for depression and tryptophan metabolism constructed.

### Protein-Protein Interaction and Molecular Docking

Interaction network for screened target proteins implicated in depression and tryptophan metabolism was constructed using String database. Top five important targets in the interaction network were selected for molecular docking. Molecular docking was used to predict interactions between protein receptors and HLJDD active compounds. Five important targets and related active compounds were selected using Dock website database for molecular docking. Molecular docking was performed by uploading the PDB ID of each target and 3D structure of active ingredients in SDF (.sdf) format. A cutoff value of 4.25 was set for the binding energy score to ensure good binding affinity of active compounds against selected targets. Binding energy scores greater than 5.0 implied stronger binding affinity. Moreover, molecular docking was carried out using AutoDock Vina (Trott and Olson, 2010). AutoDock Vina allows the ligand to be flexible whereas the target is rigid during molecular docking. Box center coordinates and size of the box were determined prior to docking experiments using. Binding energy for each active compound was determined using AutoDock Vina tool.

## Metabonomics Analysis

### Collection and Processing of Biological Samples

Mice were sacrificed with carbon dioxide immediately after behavioral assay. *Hippocampus* of each animal was isolated and frozen separately in liquid nitrogen. *Hippocampus* sample to be analyzed using LC-Q/TOF-MS was accurately weighed. ddH_2_O was added to the weighed hippocampus sample and homogenized for 3 min. 100 μL homogenate was added to ice-cold 80% methanol solution (containing 4 μg/mL L-2-chlorophenylalanine as internal standard) and vortexed for 3 min. The mixture wascentrifuged at 13,000 rpm for 15 min at 4°C. The supernatants were transferred to a centrifuge concentrator for concentration and drying. The concentrated supernatant was reconstituted with 0.1% formic acid.

### Chromatographic Conditions

A 2.1 mm × 100 mm, 2.7 μm chromatographic column was used (InfinityLab Poroshell 120 EC-C18，Agilent, United States). The mobile phase consisted of A and B, A, 0.1% formic acid in water, B, 0.1% formic acid in acetonitrile. The column temperature was set at 30°C; flow rate at 400 μL/min; injection volume at 5 μL and gradient elution conditions at 0–0.5 min, 1% B; 0.5–4 min, 1–20% B; 4–8 min, 20–100% B; 8–9 min, 100% B; 9–9.5 min, 100–1% B; 9.5–12 min, 1% **B**.

### Mass Spectrometry Conditions

Electrospray ionization source in positive mode and negative mode was used for the mass spectrometer (AB SCIEX Triple TOF™ 5600). Ionization source conditions were capillary voltage set at 3.0 kV; source temperature set at 120°C; desolvation temperature set at 350°C set at sampling cone voltage set at 30 V; ion energy at 1.0 V; cone gas flow set at 50 L/h; desolvent gas flow set at 600 L/h and mass scanning range was 100～1000 m/z.

### Data Processing and Analysis

Data were normalized using MarkerView software, and then imported into SIMCA 14.1 software. Principal component analysis (PCA) and orthogonal partial least-squares discrimination analysis (OPLS-DA) were performed after standardization. Potential different metabolites were screened using VIP >1.0, correlation coefficient |*p*(corr)| > 0.58, and those with no significant difference (*p* > 0.05) were excluded. Spectrum structure of each metabolite was obtained using HMDB database. Potential endogenous metabolites were determined using PeakView software. MetaboAnalyst 4.0 was used to predict metabolic pathways implicated in depression.

## Results

### Effect of Huang-Lian Jie-Du Decoction on Behavior of Chronic Unpredictable Mild Stress Mice as Shown by Force Swimming Test

**FIGURE 1:**
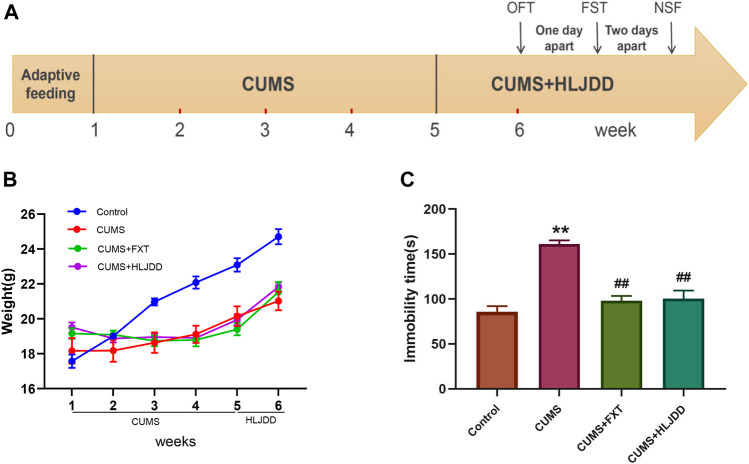
FST results showing effect of HLJDD on behavior of CUMS mice. **(A)** Schematic diagram of the experimental process; **(B)** Body weight of mice in the control group and mice in HLJDD or FXT treatment groups after six weeks (*n* = 10, Mean ± SEM); **(C)** Immobility time of mice in the control group and HLJDD or FXT treated groups after carrying out FST 1 h after treatment (*n* = 10, Mean ± SEM). *p < 0.05, **p < 0.01, compared with Control; ##*p* < 0.01, compared with CUMS via ANOVA.

Details on CUMS operation and behavior analysis are shown in Figure 1A. CUMS stressed mice showed significantly lower body weight compared weight of mice in the control group (*p* < 0.01). One-week administration of HLJDD significantly increased weight in CUMS mice (*p* < 0.01) (Figure 1B). Mice in CUMS + HLJDD group exhibited shorter immobility time after one-week treatment with HLJDD as shown by FST results (*p* < 0.01). Mice in HLJDD and FXT groups showed no significant difference in immobility time (Figure 1C). These finding imply that HLJDD is an effective antidepressant agent.

### Effect of Huang-Lian Jie-Du Decoction on Behavior of Chronic Unpredictable Mild Stress Mice as Shown by Novelty-Suppressed Feeding

**FIGURE 2:**
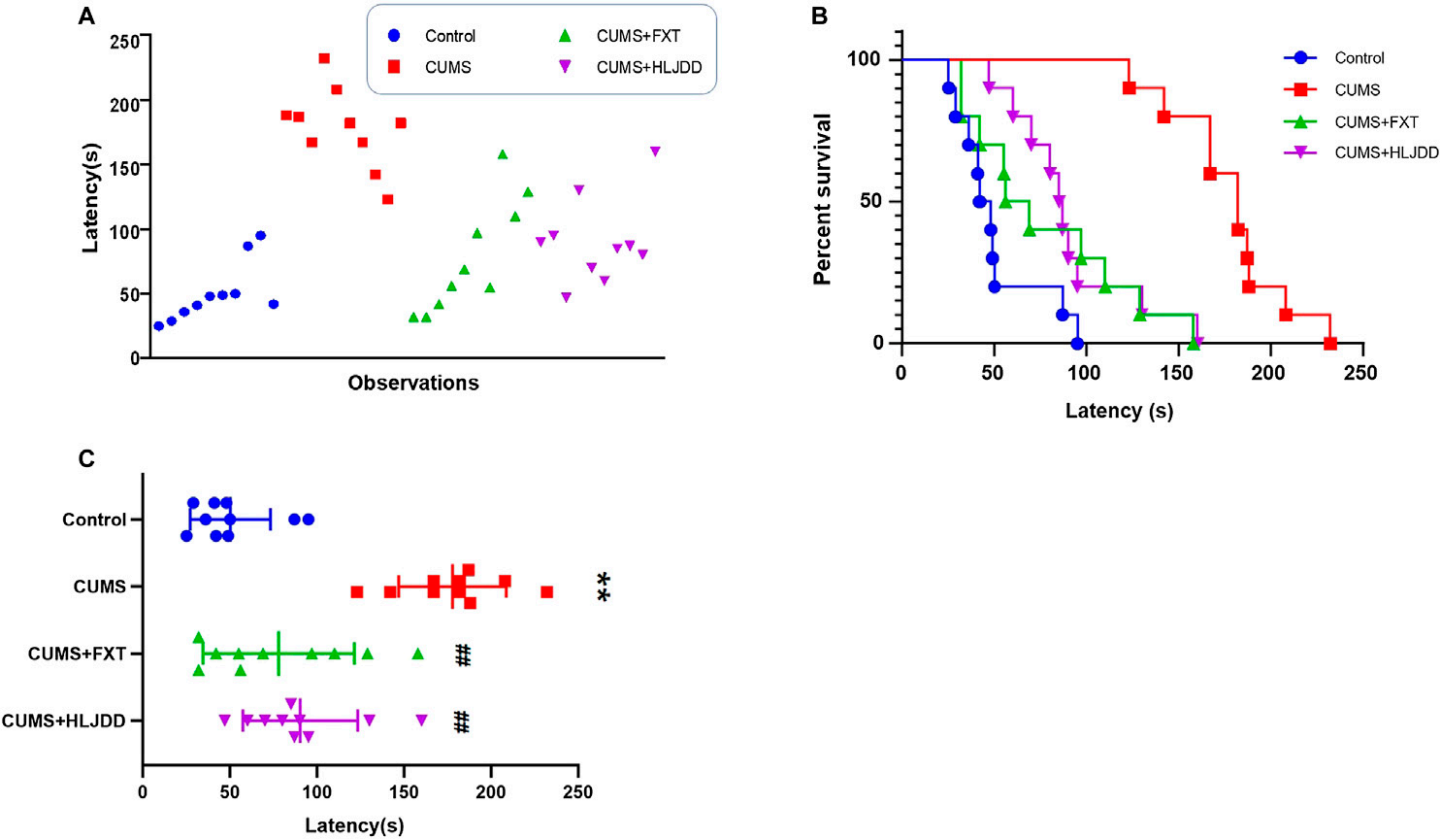
NSF results showing effect of HLJDD on behavior of CUMS mice. **(A)** Individual response times for consuming food of mice in the three groups (*n* = 10); **(B)** Kaplan–Meier curves of latency in mice in the three groups (*n* = 10); **(C)** Latency of approaching and consuming food of mice in the three groups 1 h after administration (*n* = 10, Mean ± SEM). **p < 0.01, compared with Control; ##*p* < 0.01, compared with CUMS via ANOVA.

The positive effect of HLJDD on behavior of CUMS mice shown by FST results prompted to further investigate effects of the formula on depressive-like behaviors. We conducted NSF test to determine aversion to eating by CUMS mice in a novel environment. Response times for each animal to approach and consume food are shown in Figure 2A. CUMS mice showed significantly different behaviors compared to the behavior of mice in the control and HLJDD or FXT treatment. Kaplan–Meier curves analyses demonstrated that 80% of mice in HLJDD group consumed food within 100 s. On the other hand, 80% CUMS mice without treatment consumed food in approximately 190 s (Figure 2B). Mice in HLJDD group showed significantly less time to consume food compared to mice in the control group (*p* < 0.01) (Figure 2C).

### Effect of Huang-Lian Jie-Du Decoction on Behavior of Chronic Unpredictable Mild Stress Mice as Shown by Open Field Test

**FIGURE 3:**
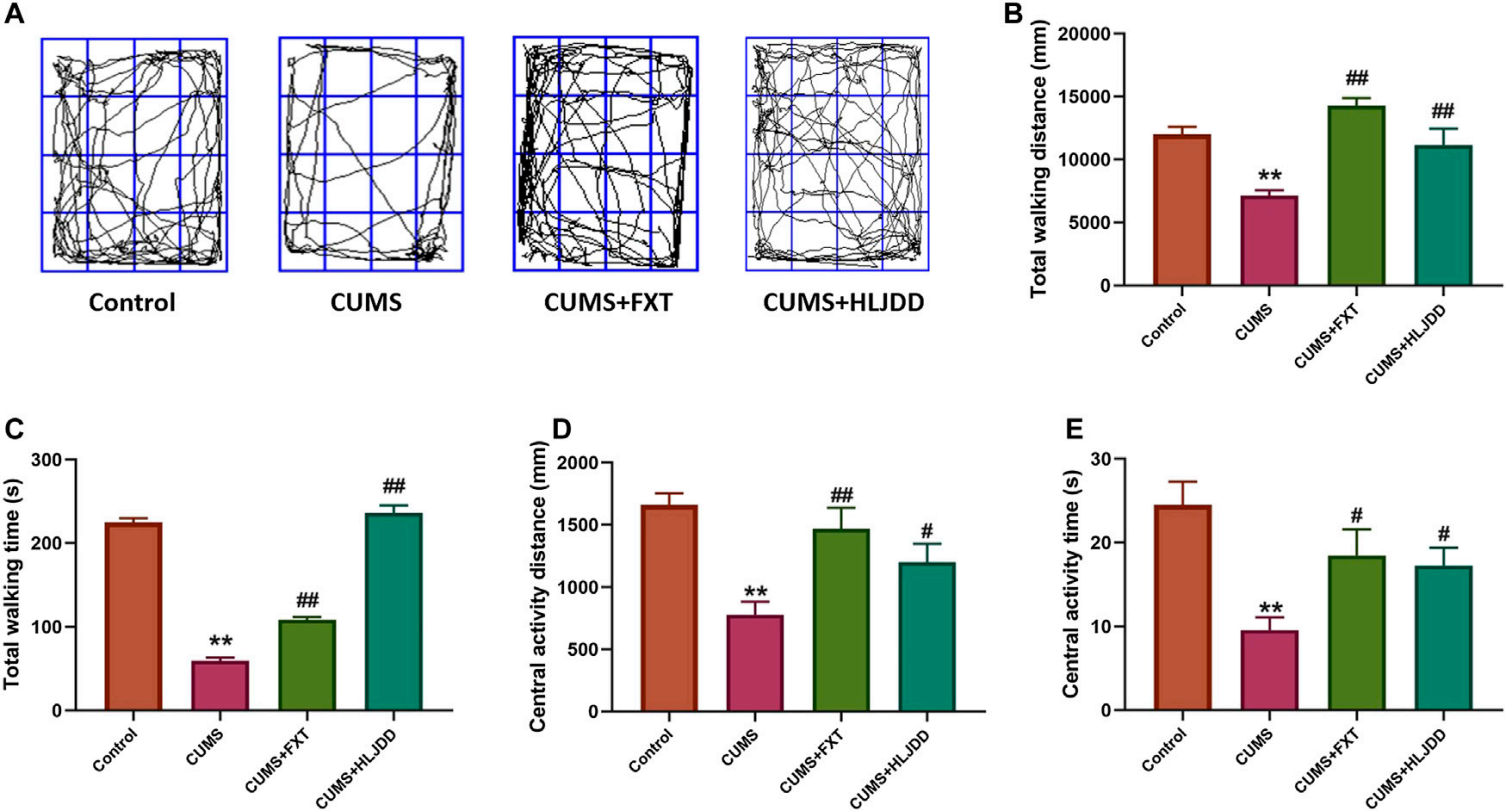
OFT results showing effect of HLJDD on behavior of CUMS mice. **(A)** Trajectory of an individual mouse over a 5-min testing session in the OFT; **(B)** Total distance covered by mice in the three groups 1 h after treatment; **(C)** Total time of movement for mice in the three groups taken 1 h before the testing; **(D)** Distance covered by mice at the center of open field in all groups 1 h after oral gavage; **(E)** Time spent by mice to move at the center of arena with or without CUMS stress and CUMS mice with the treatment of HLJDD or FXT 1 h after oral gavage. Data are expressed as Mean ± SEM, *n* = 10 mice per group. **p < 0.01, compared with Control; #*p* < 0.05, ##*p* < 0.01, compared with CUMS via ANOVA.

Mice with CUMS showed significantly shorter time and distance traveled in the center of open field. Further, OFT results showed that CUMS mice significantly shorter total movement time and distance compared to mice in the control group. HLJDD treatment improved movement time and distance taken by mice in the arena (Figure 3A). HLJDD treatment significantly increased total traveling time and distance during the 5-min session (*p* < 0.01) (Figures 3B,C). Movement trend in the center of the arena was observed. Notably, chronic HLJDD treatment significantly increased distance traveled at the center and travel duration of CUMS mice (*p* < 0.05) (Figures 3D,E).

### Effect of Huang-Lian Jie-Du Decoction on Neurotransmitters in Different Brain Regions

**FIGURE 4:**
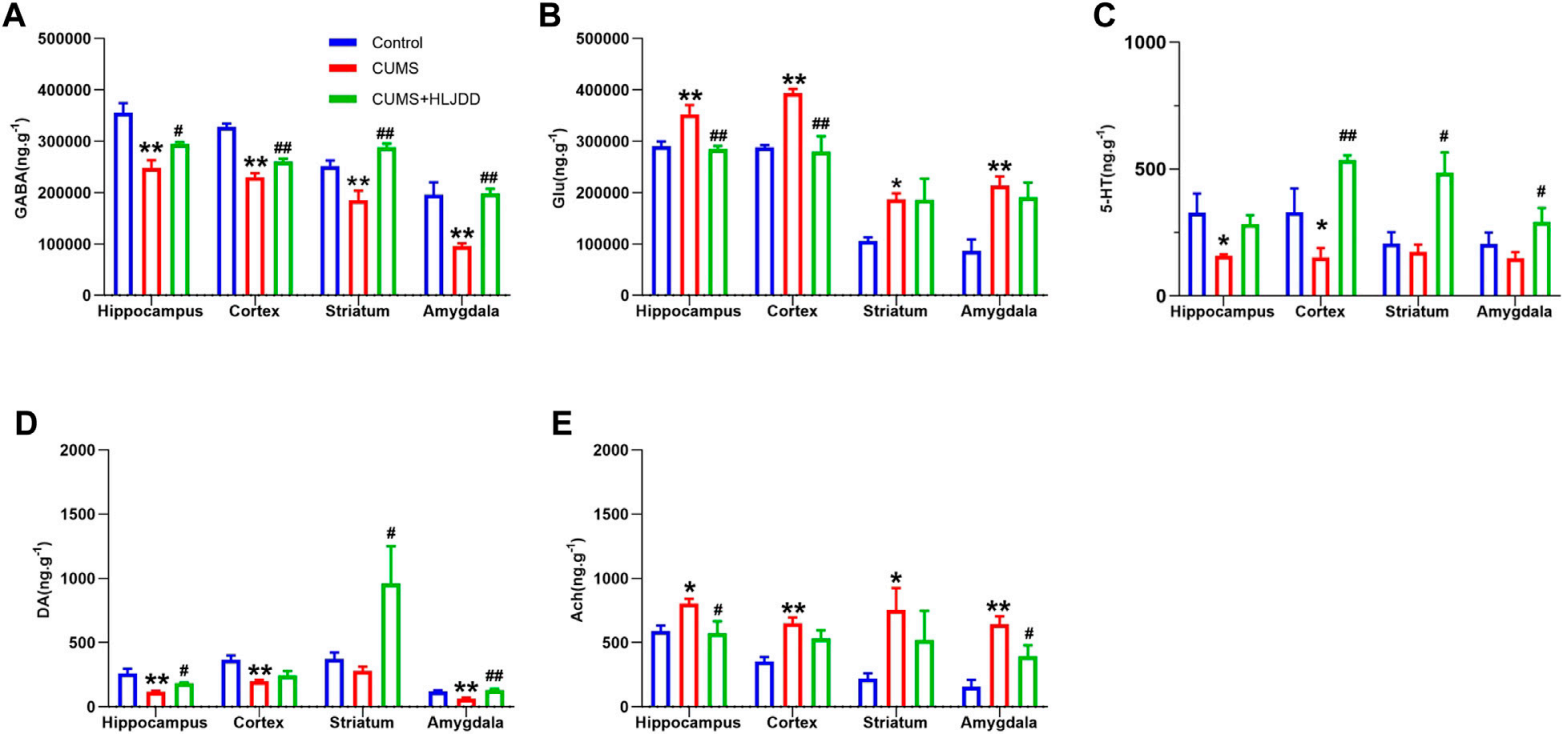
Effect of HLJDD on neurotransmitters in different brain regions. Levels of GABA **(A)**, Glu **(B)**, 5-HT **(C)**, DA **(D)**, Ach **(E)** in the hippocampus, cortex, striatum and amygdala as detected using LC-MS/MS. Data are expressed as Mean ± SEM, *n* = 10 mice per group. *p < 0.05, **p < 0.01, compared with Control; #*p* < 0.05, ##*p* < 0.01, compared with CUMS via ANOVA.

Profile of neurotransmitters in different brain regions (hippocampus, cortex, striatum and amygdala) were analyzed using LC-MS/MS. Mice in the CUMS group showed reduced levels of GABA in hippocampus, cortex, striatum and amygdala. HLJDD restored GABA levels in the four brain regions (Figure 4A). On the contrary, CUMS mice showed higher Glu levels in hippocampus, cortex striatum and amygdala and HLJDD treatment significantly decreased Glu levels in hippocampus and cortex (Figure 4B). CUMS mice showed significantly lower 5-HT levels in hippocampus and cortex compared with the levels in mice in the control group. HLJDD administration improved 5-HT levels with a significant increase observed in cortex compared with the hippocampus. Although no significant difference in 5-HT was observed in striatum and amygdala of CUMS mice prior to treatment, HLJDD administration increased 5-HT levels in these two regions (Figure 4C). HLJDD administration restored DA levels in hippocampus, cortex, striatum and amygdala of CUMS mice. HJDD treatment significantly increased DA levels in the striatum (Figure 4D). Alternatively, HLJDD administration significantly lowered Ach levels in hippocampus and amygdala (Figure 4E).

### Network Pharmacology Assay

#### Active Compounds and Targets of HLJDD on Depression and Tryptophan Metabolism

**FIGURE 5:**
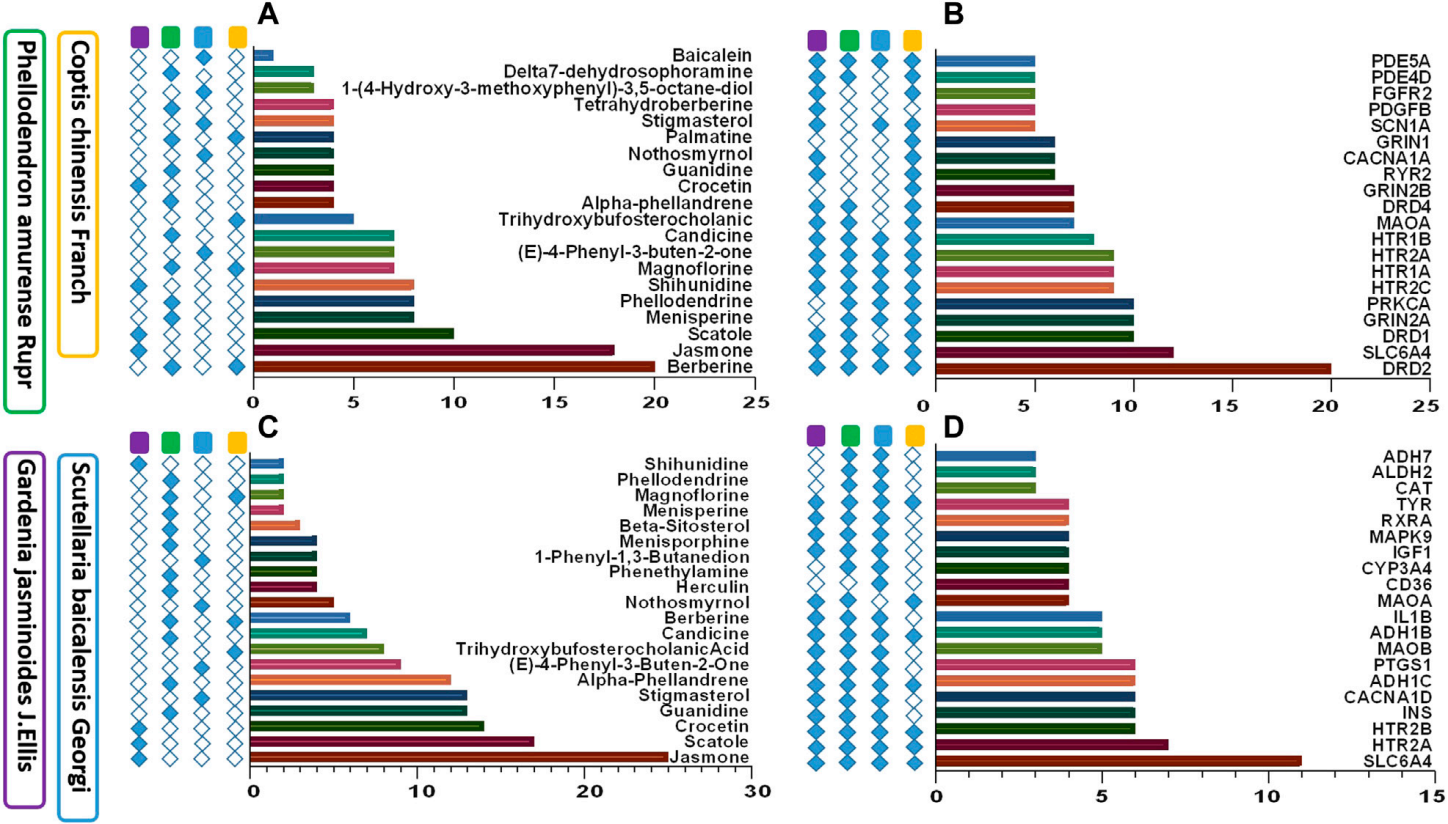
Active compounds and targets of HLJDD against depression and tryptophan metabolism. **(A)** Active ingredients of HLJDD associated with depression; **(B)** HLJDD target proteins against depression; **(C)** Active compounds of HLJDD against tryptophan metabolism; **(D)** Target proteins of HLJDD associated with tryptophan metabolism.

Area total of 1059 promising targets of HLJDD were identified after deleting duplicate targets within TCMSP, BATMAN-TCM and PharmMapper databases (161 with Coptis chinensis Franch; 684 with Scutellaria baicalensis Georgi; 612 with Phellodendron amurense Ruer, and 804 with Gardenia jasminoides J. Ellis). To explore the relationship between HLJDD and depression, 267 targets related to depressive disorders were retrieved from OMIM, TTD, and PharmGkb databases. area total of 48 common targets for HLJDD and depression were identified. Most common target proteins were D2 dopamine receptor (DRD2), Sodium-dependent serotonin transporter (SLC6A4), D1A dopamine receptor (DRD1), and Glutamate receptor ionotropic NMDA 2A (GRIN2A). A total of 67 active ingredients of HLJDD were correlated with the 48 proteins. A total of 38 active ingredients was shown to directly target the selected proteins. Common active compounds targeting the 48 proteins were Berberine, Jasmone, Skatole, Menisperine, Phellodendron, and Shihunidine. Top twenty depression-associated compounds and targets are listed in Figures 5A,B. A total of 1311 targets related to tryptophan metabolism were retrieved from OMIM, TTD, and PharmGkb databases. Out of these targets, 76 targets were common for HLJDD activity and tryptophan metabolism. Most frequently occurring target proteins were sodium-dependent serotonin transporter (SLC6A4), 5-hydroxytryptamine receptor 2A (HTR2A), 5-hydroxytryptamine receptor 2B (HTR2B) and voltage-dependent L-type calcium channel subunit alpha-1D (CACNA1D). A total of 67 active ingredients of HLJDD were implicated in binding of the selected 76 targets. Notably, a total of 37 active ingredients directly targeted the 76 target proteins. Common active ingredients against the 76 targets were Jasmone, Skatole, Crocetin, Guanidine, and Stigmasterol. These findings implied that tryptophan metabolism plays a key role in prognosis and treatment of depressive disorder. Top twenty tryptophan metabolism-associated active compounds and targets are shown in Figures 5C,D.

#### Gene Ontology Enrichment and Kyoto Encyclopedia Genes Genomes Pathways and Network Analysis of Targets Associated With Huang-Lian Jie-Du Decoction Activity on Depression

**FIGURE 6:**
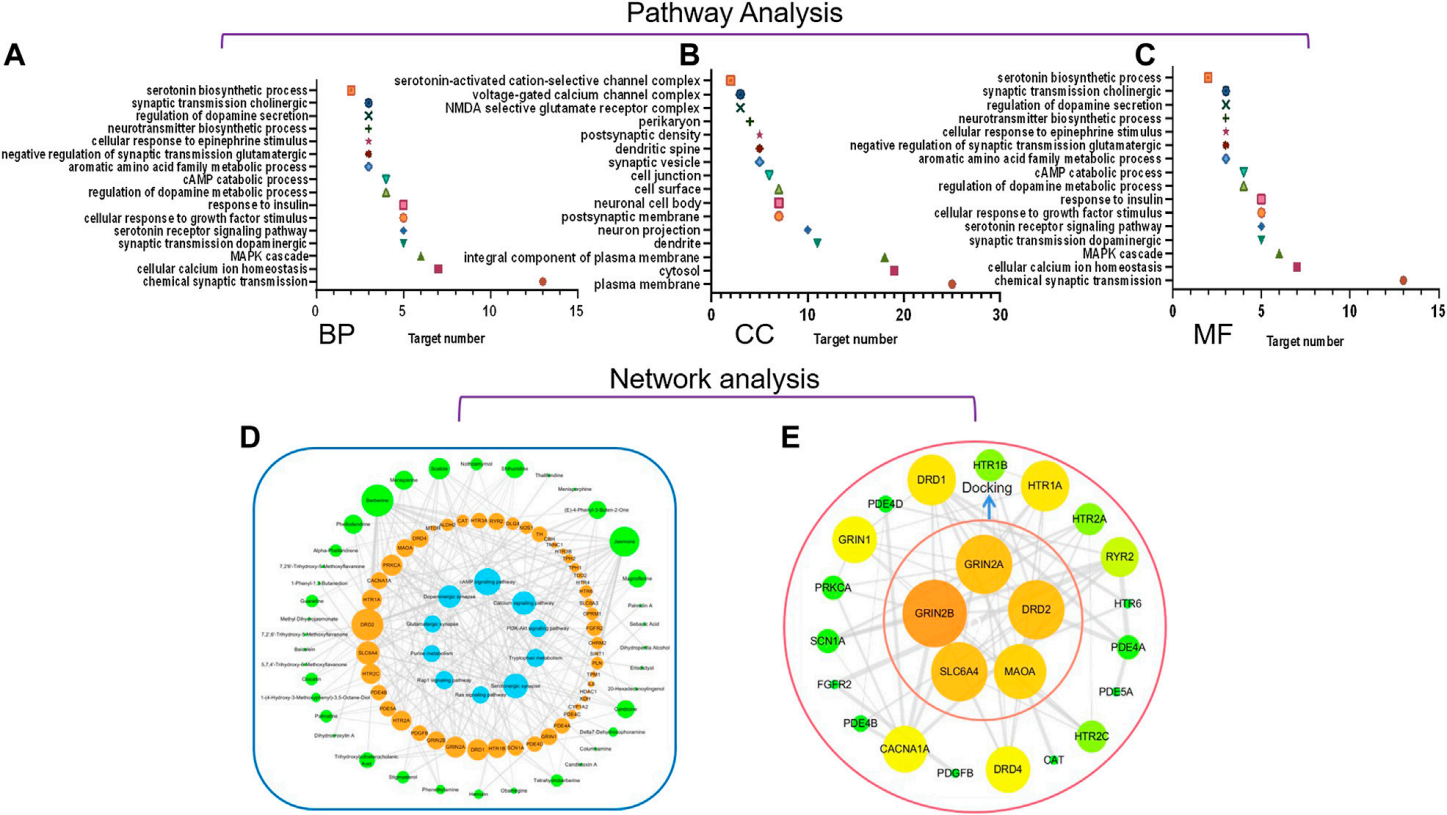
**(A–C)** GO enrichment analysis of potential targets for primary active ingredients from HLJDD against depression. BP, biological process; CC, cellular components; MF, molecular function.; **(D)** HLJDD active compound-target-pathway network of depression; **(E)** Interaction network of HLJDD target proteins involved in depression.

The 48 targets associated with HLJDD and depressive disorders were used for GO enrichment and KEGG pathway analysis using DAVID. Targets were analyzed under three levels, namely biological processes (BP), molecular function (MF) and cellular components (CC). Pathways that showed *p* < 0.05 were selected as potential pathways implicated in the activity of HLJDD against depression. Pathways identified under BP category included serotonin receptor signaling pathway, serotonin biosynthetic process, neurotransmitter biosynthetic process and aromatic amino acid family metabolic process (Figures 6A–C). Two metabolic-related pathways, five signaling-related pathways and three synaptic-related pathways were identified suing In the KEGG pathway assay. These pathways are implicated in serotonergic synaptic and tryptophan metabolism pathway.

A compound-target-pathway network for active compounds in HLJDD implicated in treatment of depression was constructed using Cytoscape software (Figure 6D). The network showed 96 nodes with 48 of these nodes presented in orange. A larger the circle implied more active compounds and key pathways associated with depression. A total of 38 active ingredients were indicated in green, and 10 disease pathways were indicated in blue. Active compounds and pathways formed a complete network through 490 edge connections. Potential relationships between active compounds and targets or targets and pathways were expressed by edges. The degree represented the size of nodes, with a greater degree implying that the node plays a role in modulation of depression by HLJDD. Several active compounds were implicated in DRD1, DRD2, SLC6A4, and GRIN2A. Key pathways where these targets are involved in are cAMP signaling pathway, calcium signaling pathway, serotonergic synaptic pathway and dopaminergic synaptic pathway. Berberine had the most interactions as it interacted with twenty proteins and ten pathways.

In this study, interactions between anti-depressant targets and HLJDD active compounds were analyzed using String database. Interaction network of anti-depressant targets and HLJDD active compounds was constructed based on the degree value greater than medium of ranking. The network consisted of 24 nodes and 134 edges. Results were saved in TSV format and imported into Cytoscape for identification of key targets (Figure 6E). Higher degrees on nodes such as GRIN2B, GRIN2A, DRD2, SLC6A4, and MAOA implied that they play an important role in antidepressant activity.

#### Pathways and Network Analysis Huang-Lian Jie-Du Decoction Targets Associated With Tryptophan Metabolism

We further explored interaction network between HLJDD and tryptophan metabolism as tryptophan metabolism plays a key role in pathological process of depression. GO enrichment analysis and KEGG pathway analysis of the 76 targets involved in tryptophan metabolism was carried using DAVUD database. Pathways in which targets were implicated in were classified into three levels: BP, MF, and CC and those pathways with *p* < 0.05 were selected. Under the BP category pathways involved in drug response, regulation of insulin secretion and inflammatory response were identified. A total of 16 tryptophan metabolism-related pathways (*p* < 0.05) were screened in five categories using KEGG pathway analysis. Three pathways were metabolic-related pathways, nine were signaling-related pathways, two were synaptic-related pathways, one was a disease-related pathway and one inflammation pathway.

**FIGURE 7:**
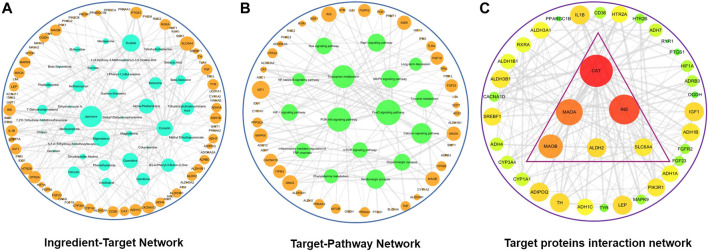
Tryptophan metabolism network diagrams after HLJDD treatment. **(A)** HLJDD active ingredient-target network associated with tryptophan metabolism; **(B)** HLJDD target-pathway network associated with tryptophan metabolism; **(C)** Core HLJDD target proteins interaction network associated with tryptophan metabolism.

Compound-target network for tryptophan metabolism was constructed using Cytoscape based on active compounds and predicted target. The network had a total of 113 nodes. Out of these nodes, 37 were active compounds expressed in blue and 76 were targets expressed in orange. Active compounds and targets were connected by 372 edges. Main targets identified were SLC6A4, HTR2A, HTR2B, and INS which were mainly targeted by Jasmone, Skatole, berberine. SLC6A4 had the highest degree value among the selected targets (Figure 7A). In addition, a target-pathway network map of 92 nodes was constructed using Cytoscape. This network showed a total of 76 targets expressed in orange and 16 pathways expressed in green. 256 connecting edges formed the network. SLC6A4, HTR2A, HTR2B, and INS targets are involved in serotonergic synaptic pathway, FoxO signaling pathway and inflammatory mediator regulation of TRP channels pathway (Figure 7B).

Interaction network of tryptophan metabolism targets and HLJDD active compounds was constructed using a degree value greater than the medium target. The network consisted of 38 nodes and 326 edges. Results were saved in TSV format and imported into Cytoscape software for selection of key targets (Figure 7C). Nodes with the highest degree represented targets that played an important role in the network.

#### Huang-Lian Jie-Du Decoction Active Compounds Target Proteins Implicated in Depression

Five important depression targets identified using the network map (GRIN2B, GRIN2A, DRD2, SLC6A4, and MAOA) and twenty-one related active compounds were selected for molecular docking. The PDB-ID for each target was retrieved from RCSB PDB database. The largest search term match score was selected as the optimal PDB-ID value. 3D structural formula of the active compounds were retrieved from Pubchem database. Four pair ingredient-target docking scores were greater than 7.0 and accounted for 3.81%. Seventy-one docking scores were between 7.0 and 5.0 and accounted for 67.62%. Further, sixteen docking scores were between 5.0 and 4.25 and accounted for 15.24% whereas fourteen docking scores were less than 4.25 and accounted for 13.33%. Five tryptophan metabolism targets including CAT, INS, MAOA, MAOB, ALDH2, and SLC6A4 and eight related active compounds were selected for molecular docking. Docking results showed five docking scores greater than 7.0 which accounted for 12.50%; twenty-five docking scores between 7.0 and 5.0 which accounted for 62.50%; four docking scores between 5.0 and 4.25 which accounted for 10.00% and six docking scores less than 4.25 which accounted for 15.00%.

**FIGURE 8:**
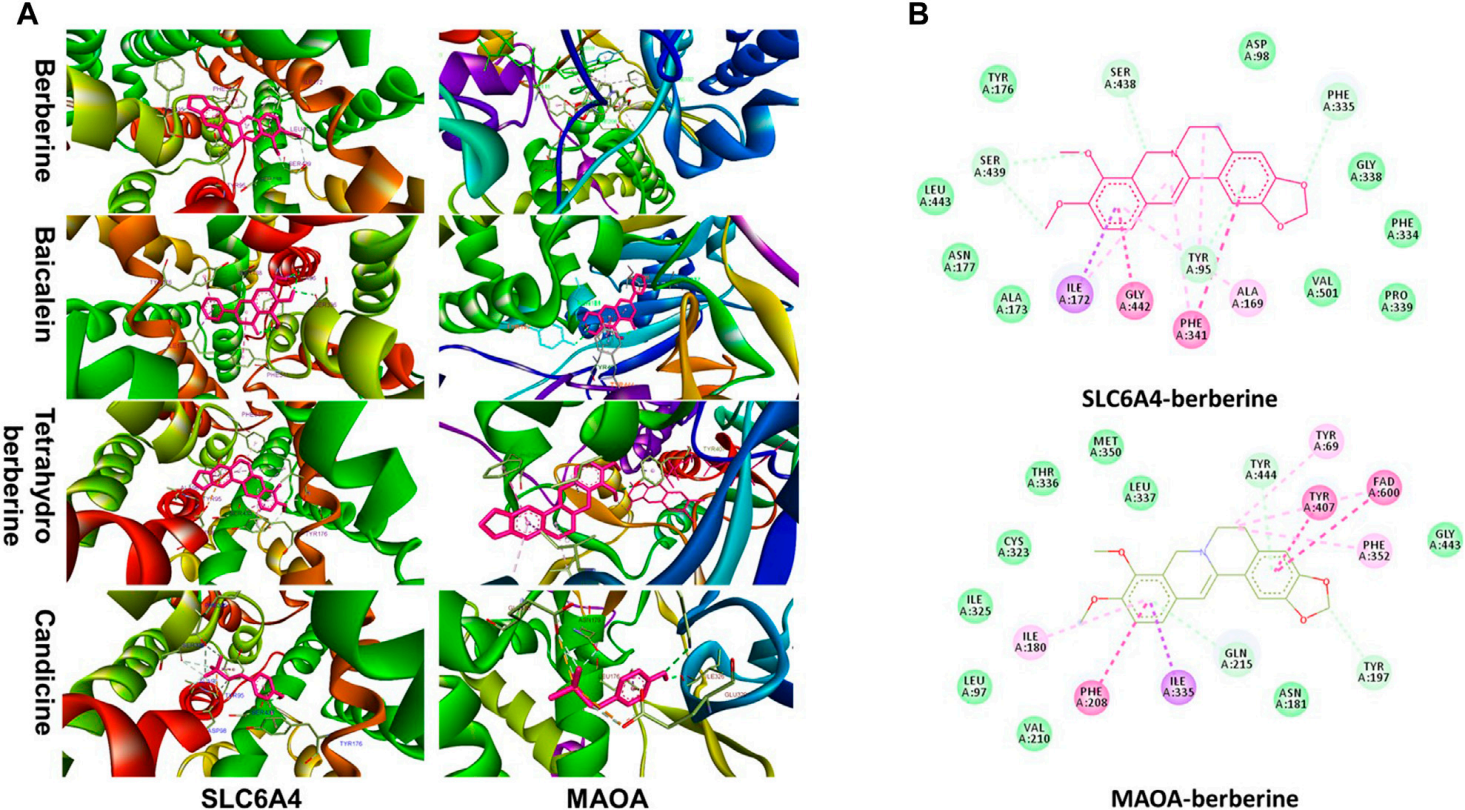
**(A)** Stereoview of substrate/inhibitor binding of SLC6A4 and MAOA with berberine, baicalein, tetrahydroberberine, and candicine; **(B)** Biding poses of berberine at SLC6A4 and MAOA binding sites.

Further molecular docking analysis was performed between SLC6A4, MAOA and their predicted ligands berberine, baicalein, tetrahydroberberine, candicine using AutoDock Vina. Binding energy between SLC6A and baicalein, berberine, tetrahydroberberine, and candicine were −10.1, −10, −8.8, and −6.5 kcal mol^−1^, respectively for Binding energy scores of berberine, baicalein, tetrahydroberberine, and candicine against MAOA were −8.4, −8.4, −7.6, −6.2 kcal mol^−1^ (Figure 7). Berberine binds at the catalytic cavity of SLC6A4 and interacts with 17 amino acid residues. The interaction between berberine and Try-95 was a hydrogen bond. Moreover, berberine interacts with Try-176 through van der Waals force and Gly 342 and Phe-341 through a π–π interaction. Berberine interacts with 19 amino acid residues at the active site of MAOA. with the interaction between berberine and Tyr-197 was a hydrogen bond whereas interactions with Tyr-407, Fad-600 and Phe-208 were π–π interactions (Figure 8). A 2D representation the binding poses and interactions between SLC6A4 and MAOA, and baicalein, tetrahydroberberine and candicine.

#### Hippocampal Metabolomics Analysis

LC-Q/TOF-MS metabolic profile data were analyzed using PCA in unsupervized mode. Data for the groups were completely separated within the two modes, ESI^+^ and ESI^−^. This implied that metabolic profile of hippocampal tissue for CUMS mice treated with HLJDD was significantly from the profile for untreated mice. To further identify the different metabolites, we used supervised mode OPLS-DA to reduce effect of intra-group differences on classification thus maximizing separation between groups. 200 permutation validations were performed to verify reliability of the model and prevent overfitting. All Q2 values on the left were lower than values on the right. The intersection value between the regression line of Q2 and the longitudinal axis was less than zero. In summary, the model was accurate in prediction of metabolites.

**FIGURE 9:**
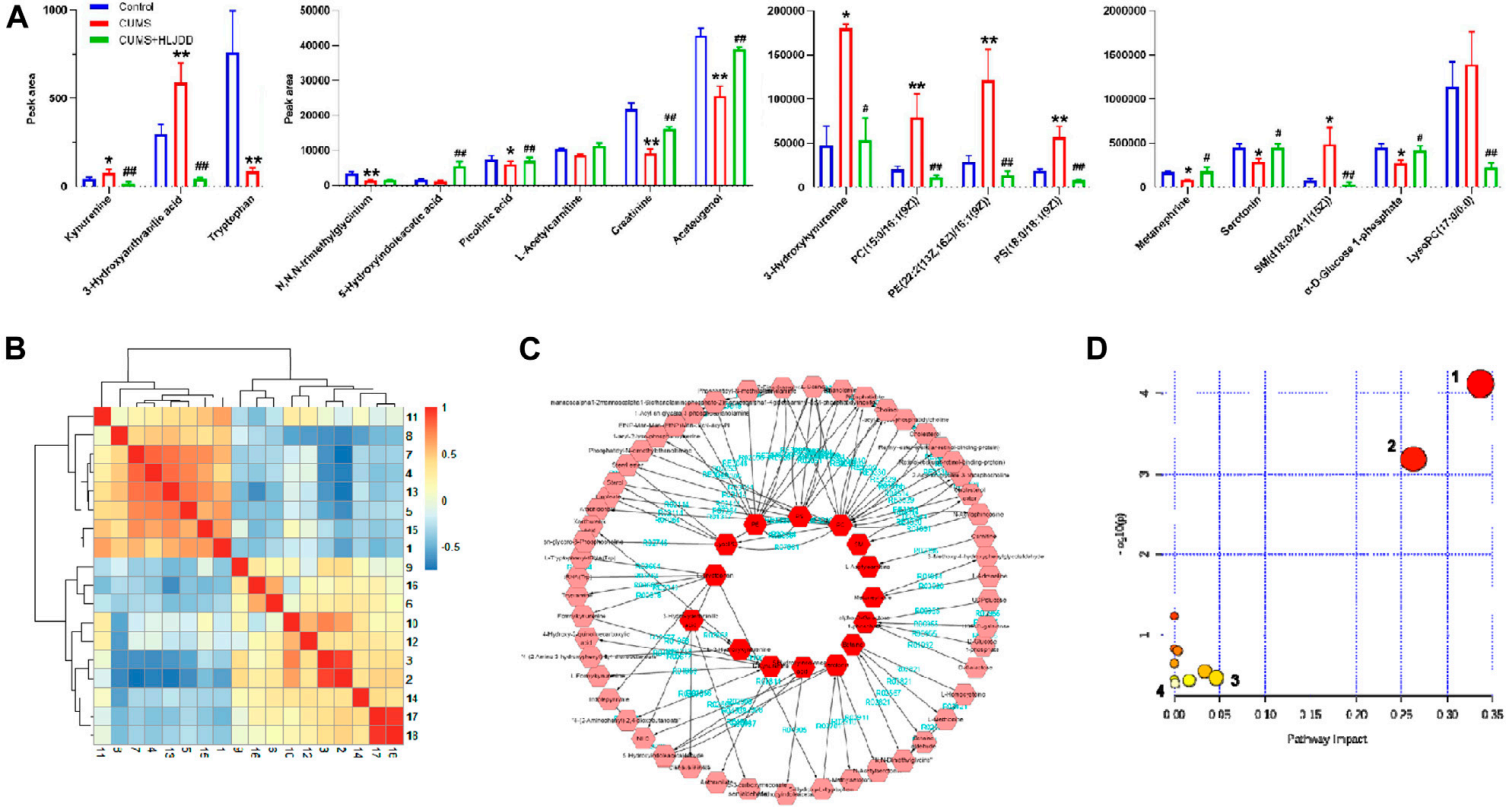
Metabonomics analysis of hippocampus from CUMS mice using LC-Q/TOF-MS. **(A)** Metabolites associated with CUMS conditions with or without HLJDD; **(B)** Correlation heatmap of the 18 different metabolites; **(C)** Metabolic network of the metabolites constructed using MetScape; **(D)** Metabolic pathways as visualized using MetaboAnalyst. 1, tryptophan metabolism; 2, glycerol phospholipid metabolism; 3, glycine, serine and threonine metabolism; 4, tyrosine metabolism. Data are expressed as Mean ± SEM, *n* = 10 mice per group. *p < 0.05, **p < 0.01, compared with Control; #*p* < 0.05, ##*p* < 0.01, compared with CUMS via ANOVA.

VIP≥1 in OPLS-DA mode, |*p*(corr)| > 0.58 in S-Plot mode was used as the screening criteria for exploring different metabolites involved in depression using ANOVA (*p* < 0.05). A total of 18 different metabolites were identified based on secondary spectrum information and HMDB database in which 8 metabolites were up-regulated and 10 metabolites were down-regulated. Variation of the 18 metabolites was reversed by administration of HLJDD (Figure 9A). Kynurenic metabolism process involved in tryptophan metabolism was inhibited by HLJDD compounds by inhibition of kynurenine, 3-hydroxykynurenine and 3-hydroxyanthranilic acid. On the contrary, serotonin metabolism was promoted by HLJDD by increasing serotonin and 5-hydroxyindoleacetic acid.

**FIGURE 10:**
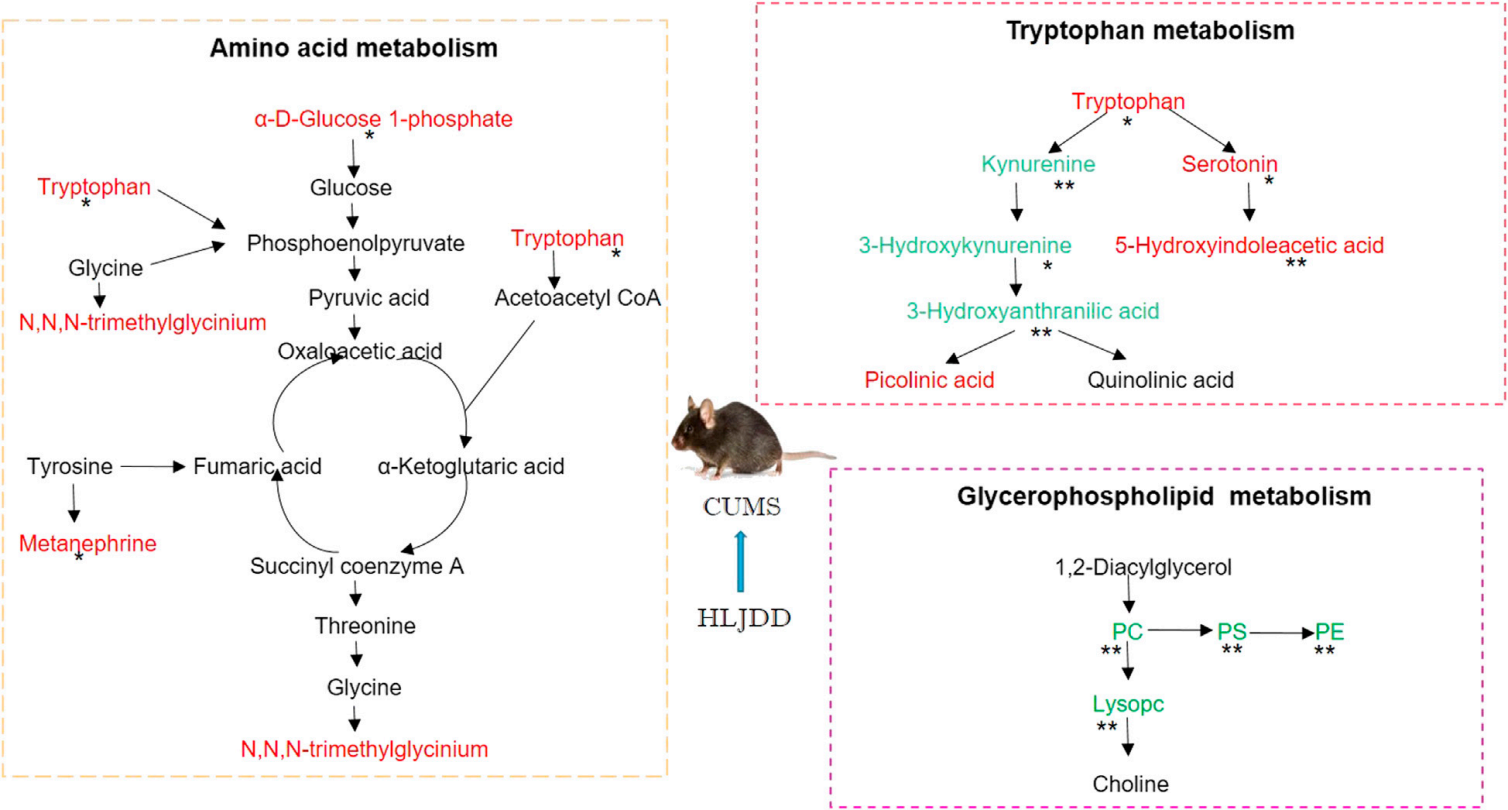
Representative metabolic pathways demonstrated in metabonomics analysis, green represents pathways downregulated by HLJDD whereas red represents pathways upregulated by HLJDD.

Correlation analysis of 18 different metabolites were carried out and results presented as a heatmap using R software V3.6.3. Metabolites involved in serotonin metabolism (tryptophan, 5-hydroxyindoleacetic acid, tryptophan) showed a positive correlation with metabolites involved in kynurenine metabolism (kynurenine, 3-hydroxykynurenine, 3-hydroxyanthranilic acid). Serotonin metabolism showed a negative correlation with kynurenine metabolism was observed (Figure 9B). This implies that HLJDD promotes kynurenine metabolism and inhibits serotonin metabolism. MetScape, a Cytoscape plug-in was used to construct a composite network related to drug efficacy to further explore the mechanism of action of HLJDD active compounds. In the network, selected biomarkers were represented by a red hexagon, whereas metabolic reactions were represented by lines between the metabolites. The metabolic network consisted of 68 nodes and 110 edges (Figure 9C). The network shows internal connection between endogenous substances and presents antidepressant effect of HLJDD. Identified biomarkers were used to explore depression related metabolic pathways, including tryptophan metabolism, glycerol phospholipid metabolism, glycine, serine and threonine metabolism and tyrosine metabolism, using MetaboAnalyst database. (Figure 9D). Main metabolic pathways are shown in Figure 10.

## Discussion

Mood disorders, including major depressive disorder (MDD) and bipolar disorder (BD), are the most prevalent psychiatric conditions worldwide. World Health Organization predicted that MDD will be the leading cause of disease burden by 2030 (Reddy, 2012). Although neurological systems play a key role in development of MDD, the mechanism of action of specific neurological systems at onset, relapse and treatment of MDD are not fully known. Further, available antidepressant medications acting on monoaminergic systems are not effective. Therefore, poor understanding of pathophysiological process of MDD and lack of effective therapies limit attempts to eliminate MDD. The findings of this study imply that. There is need to come up with alternatives for treatment of MDD future. In addition, novel strategies should be designed to develop alternative MDD therapies. Further, studies should explore whether there are key pathways and targets implicated in pathogenesis and treatment of MDD in addition to tryptophan metabolism.

Herbal medicines have seen increased interest as promising complementary and alternative therapies for depressive disorders. Medicinal plants have been in use for several years as they have multiple active ingredients. Herbal medicines are widely adopted as therapy approaches against minor and mild depressive symptoms worldwide. *Hypericum* (St. John’s wort, United Kingdom; Johanniskraut, Germany) showed significantly higher activity compared with placebo in the treatment of depression using Hamilton Depression Rating Scale. *Hypericum* mediates antidepressant effect by inhibiting reuptake of 5-HT, NA, and DA into the presynaptic neurons (Calapai et al., 2001). Other as antidepressant preparations inhibit transient receptor potential cation channel 2 channels and voltage-gated calcium channel-dependent Ca^2+^ mobilization, inhibit oxidative stress, and have anti-inflammatory activities against MDD related symptoms (Naziroglu et al., 2014). Herbal preparations have a myriad of pharmacological actions due to diversity of bioactive compounds (Jakovljevic et al., 2000). In China, herbal medicine has been in use for more than 8,000 years is increasingly been introduced for depressive treatment (Liu et al., 2015). Araliaceae elevates levels of 5-HT, NE, and DA in the brain, and upregulates CREB protein level (Jin et al., 2013). Antidepressant effects of Panax ginseng are mediated through modulation of monoamine neurotransmitters and neurotrophic factors, regulating the function of HPA axis, and anti-inflammatory activity (Jin et al., 2019). Herbal formula with several herbal medicines modulates more targets compared with single compound herbal preparation. Previous studies report activities of TCM formula such as Danzhi Xiaoyao powder, Gengnianchun, and Yixin Ningshen tablet against depression (Wu et al., 2020; Zhang et al., 2020; Zhu et al., 2020). The findings of our study show that HLJDD improves depressive disorders primarily through modulation of tryptophan metabolism, dopaminergic synaptic function, and cAMP, FoxO and calcium signaling pathways. Beside those pleiotropic pharmacological actions, herbal preparations and TCM formula have less side effects and are relatively affordable. Studies have shown that dysregulation or dysfunction of depression-associated metabolic or biochemical pathways play a key role in pathogenesis of depression. These findings form a basis for development of novel therapeutic approaches such as cocktail medicines, refined herbal medicines with multiple targets to inhibit the various pathways involved.

Multi-targets therapeutics are promising approaches for management of depressive disorders. Current drug design approaches are mainly target-based focusing on single target implicated in disease. Unfortunately, new candidates targeting first-in-class drug targets are less effective than first generation drugs and have more side effects. Moreover, discovery and development of novel molecular targets is a challenging and a slow process. Development of multi-target therapeutics will help overcome limitations of monotherapies through modulation of multiple cellular pathways (Borisy et al., 2003; Keith et al., 2005). Duloxetine (Cymbalta®) treats depression through inhibition of serotonin and norepinephrine reuptake. Brexpiprazole, a second-generation antidepressant, was approved by FDA in 2015 for treatment of MDD. As a novel antidepressant medicine, Brexpiprazole achieves its activity by modulating serotonin-dopamine activity. It partially activates dopamine D2 and serotonin 5HT1A receptors. Additionally, it has potent antagonistic activity against serotonin 5HT2A, noradrenergicα1B and α2C receptors (Beyer and Weisler, 2016). Multi-target therapies can be achieved through 3 approaches; 1) medicine cocktails acting on different targets that are involved in separate pathophysiological process or various nodes of the same signaling pathway; 2) one medicine with multicomponents delivered through a single system acting on the same target such as Synercid^®^ (Manfredi and Sabbatani, 2010); 3) a single compound acting on multi-target. Exploration of the relationship between multiple ligands and multi-target through network pharmacology analysis is important in development of new drug candidates. The strategy is widely applicable for development of herbal medicines. In our study, HLJDD formula showed multi-target effects as an antidepressant (Figure 8). Dopamine D2 and D4 receptors of dopaminergic synapse and serotonin 5HT1A, 5HT2A, and 5HT4 receptors of serotonergic synapse are HLJDD targets for treatment of depressive disorder. Moreover, MAOA enzyme and SLC6A4 transporter associated with tryptophan metabolism play key role in mediating activity of HLJDD against depression. HLJDD consists of berberine, baicalein, tetrahydroberberine, and candicine compounds that target SLC6A4 and MAOA with good binding energy scores. Pharmacological activity of HLJDD against depression is due to multiple compound properties and multi-target action.

Currently, monoamine hypothesis of depression is the leading theory guiding drug discovery and clinical practice. The theory suggests assay of serotonin level in plasma and cerebrospinal fluid for diagnosis of depression (Perez-Caballero et al., 2019). First generation of antidepressant medicines modulate serotonin levels in synaptic space by inhibiting monoamine oxidase activity or neurotransmitter transporters. Follow-up antidepressants restore serotonin levels in synaptic cleft by selectively inhibiting serotonin reuptake and selective inhibition of noradrenaline reuptake (Jans et al., 2007). Current antidepressants target 5-hydroxytryptamine (5-HT) receptors. These drugs are either agonists or antagonists which are more effective than previous drugs and have rapid onset activity. Different 5-HT receptor subtypes play different roles in modulating mood, memory, sleep, appetite and circadian rhythms. 5-HT1A receptor antagonist and 5-HT4 receptor agonist increase cAMP levels and produce behavioral and neurochemical antidepressant-like effects. Canonical antidepressants have slow and moderate effects which can be overcome by modulators of glutamate receptor and 5-HT4R which have rapid and long-lasting antidepressant effects (Rosel et al., 2004; Valentine et al., 2011). Traditional antidepressants are characterized by withdraw and/or persistent side effects following long-term administration therefore, there is need to develop alternative pharmacological and non-pharmacological interventions. The findings of this study show that HLJDD modulates tryptophan metabolism and translocation. In addition, active compounds in HLJDD modulates activities of the serotonin receptors 5HT1A, 5HT2A, and 5HT4. Therefore, HLJDD mediates antidepressant activities through modulation of tryptophan metabolism and serotonergic synapse function.

An interaction network for depression-associated targets and corresponding compounds in HLJDD showed that several pathways are implicated in activity of HLJDD against MDD. In addition to tryptophan metabolism process and receptor modulation, dopamine receptors DRD2 and DRD4 are involved in therapeutic effects of HLJDD. Insulin signaling associated with glucose metabolism in neurons was identified, which is consistent with findings that intravenous insulin improves mood and cognition, and depression subjects have glucose disruption (Kozumplik and Uzun, 2011; Strachan, 2005). Although multi-targets actions mediate polypharmacology of HLJDD on depression disorder, action intensity and dynamics process on these targets should be explored through *in vitro* and *in vivo* experiments. Regulation of tryptophan metabolism by HLJDD is mediated through serotonin metabolism and kynurenine metabolism. Although, active phytochemicals were selected based on drug-like rules, novel available delivery system should be explored to improve bioavailability of compounds to specific brain regions. More studies on development of a multi-component therapy from HLJDD and explore different key targets for treatment of depression.

## Conclusion

HLJDD, a TCM formula showed significant therapeutic effects on depressive disorder. HLJDD ameliorated depression-like behavior of CUMS mice using FST, NSF and OFT assays. Multiple pharmacological actions of HLJDD on depression are mainly achieved by modulation of tryptophan metabolism, monoamine nervous system function and energy metabolism. HLJDD provides scaffolds for development of a novel alternative antidepressant therapies.

## Data Availability

The original contributions presented in the study are included in the article/Supplementary Material, further inquiries can be directed to the corresponding author/s.

## Glossary

5-HTSerotonin

5-HPT5-hydroxytryptophan

Achacetylcholine

ADAlzheimer’s disease

ADME/Tabsorption, distribution, metabolism, excretion and toxicity

BPbiological process

CACNA1DVoltage-dependent L-type calcium channel subunit alpha-1D

CCCellular ingredients

CUMSChronic unpredictable mild stress

DADopamine

DLDrug-likeness

DRD1D1A dopamine receptor

DRD2D2 dopamine receptor

EEpinephrine

ECEnterochromaffin cells

FXTFluoxetine HCL

FSTForce swimming test

GABAγ-aminobutyric acid

Gluglutamic acid

GRIN2Aglutamate receptor ionotropic NMDA 2 A

HLJDDhuang-lian Jie-du Decoction

HTR2A5-hydroxytryptamine receptor 2A

HTR2B5-hydroxytryptamine receptor 2B

IDOindolenine 2,3 dioxygenase

MAOIsmonoamine oxidase inhibitors

MFmolecular function

NEnorepinephrine

NSFnovelty-suppressed feeding test

OBoral bioavailability

SLC6A4sodium-dependent serotonin transporter

SSRIsselective serotonin reuptake inhibitors

TCAtricyclic antidepressants

## References

[B1] Bai M. Zhu X. Zhang Y. Zhang S. Zhang L. Xue L. (2012). Abnormal hippocampal BDNF and miR-16 expression is associated with depression-like behaviors induced by stress during early life. PLoS One 7, e46921. 10.1371/journal.pone.0046921 23056528 PMC3466179

[B2] Bang-Andersen B. Ruhland T. Jorgensen M. Smith G. Frederiksen K. Jensen K. G. (2011). Discovery of 1-[2-(2,4-dimethylphenylsulfanyl)phenyl]piperazine (Lu AA21004): a novel multimodal compound for the treatment of major depressive disorder. J. Med. Chem 54, 3206–3221. 10.1021/jm101459g 21486038

[B3] Beyer J. L. Weisler R. H. (2016). Adjunctive brexpiprazole for the treatment of major depressive disorder. Expet Opin. Pharmacother. 17, 2331–2339. 10.1080/14656566.2016.1254188 27788337

[B4] Borisy A. A. Elliott P. J. Hurst N. W. Lee M. S. Lehar J. Price E. R. (2003). Systematic discovery of multicomponent therapeutics. Proc. Natl. Acad. Sci. U.S.A 100, 7977–7982. 10.1073/pnas.1337088100 12799470 PMC164698

[B5] Calapai G. Crupi A. Firenzuoli F. Inferrera G. Squadrito F. Parisi A. (2001). Serotonin, norepinephrine and dopamine involvement in the antidepressant action of hypericum perforatum. Pharmacopsychiatry 34, 45–49. 10.1055/s-2001-15180 11302563

[B6] Chouinard G. Chouinard V. A. (2015). New classification of selective serotonin reuptake inhibitor withdrawal. Psychother. Psychosom. 84, 63–71. 10.1159/000371865 25721565

[B7] Cipriani A. Furukawa T. A. Salanti G. Chaimani A. Atkinson L. Z. Ogawa Y. (2018). Comparative efficacy and acceptability of 21 antidepressant drugs for the acute treatment of adults with major depressive disorder: a systematic review and network meta-analysis. Focus 16, 420–429. 10.1176/appi.focus.16407 32021580 PMC6996085

[B8] Dai Y. Sun L. Qiang W. (2018). A new strategy to uncover the anticancer mechanism of Chinese compound formula by integrating systems pharmacology and bioinformatics. Evid Based Complement Alternat Med 2018, 6707850. 10.1155/2018/6707850 30108661 PMC6077598

[B9] Dulawa S. C. Hen R. (2005). Recent advances in animal models of chronic antidepressant effects: the novelty-induced hypophagia test. Neurosci. Biobehav. Rev. 29, 771–783. 10.1016/j.neubiorev.2005.03.017 15890403

[B10] Fava G. A. (2003). Can long-term treatment with antidepressant drugs worsen the course of depression?. J. Clin. Psychiatr. 64, 123–133. 10.4088/jcp.v64n0204 12633120

[B11] Freis E. D. (1954). Mental depression in hypertensive patients treated for long periods with large doses of reserpine. N. Engl. J. Med. 251, 1006–1008. 10.1056/NEJM195412162512504 13214379

[B12] Ge Q. Chen L. Yuan Y. Liu L. Feng F. Lv P. (2020). Network pharmacology-based dissection of the anti-diabetic mechanism of Lobelia chinensis. Front. Pharmacol. 11, 347. 10.3389/fphar.2020.00347 32265717 PMC7099657

[B13] Guo X. Qiu W. Liu Y. Zhang Y. Zhao H. Chen J. (2017). Effects of refined xiaoyaosan on depressive-like behaviors in rats with chronic unpredictable mild stress through neurosteroids, their synthesis and metabolic enzymes. Molecules 22, 1386. 10.3390/molecules22081386 PMC6152155 28825678

[B14] Jakovljevic V. Popovic M. Mimica-Dukic N. Sabo A. Gvozdenovic L. (2000). Pharmacodynamic study of *Hypericum perforatum* L. Phytomedicine 7, 449–453. 10.1016/S0944-7113(00)80027-6 11194172

[B15] Jans L. A. Riedel W. J. Markus C. R. Blokland A. (2007). Serotonergic vulnerability and depression: assumptions, experimental evidence and implications. Mol. Psychiatr. 12, 522–543. 10.1038/sj.mp.4001920 17160067

[B16] Jin L. Wu F. Li X. Li H. Du C. Jiang Q. (2013). Anti-depressant effects of aqueous extract from Acanthopanax senticosus in mice. Phytother Res. 27, 1829–1833. 10.1002/ptr.4938 23418105

[B17] Jin Y. Cui R. Zhao L. Fan J. Li B. (2019). Mechanisms of Panax ginseng action as an antidepressant. Cell Prolif 52, e12696. 10.1111/cpr.12696 31599060 PMC6869450

[B18] Keith C. T. Borisy A. A. Stockwell B. R. (2005). Multicomponent therapeutics for networked systems. Nat. Rev. Drug Discov. 4, 71–78. 10.1038/nrd1609 15688074

[B19] Kozumplik O. Uzun S. (2011). Metabolic syndrome in patients with depressive disorder–features of comorbidity. Psychiatr. Danub. 23, 84–88. 10.1055/s-0030-1248553 21448104

[B20] Lee B. Sur B. Yeom M. Shim I. Lee H. Hahm D. H. (2012). Effect of berberine on depression- and anxiety-like behaviors and activation of the noradrenergic system induced by development of morphine dependence in rats. Korean J. Physiol. Pharmacl. 16, 379–386. 10.4196/kjpp.2012.16.6.379 PMC3526741 23269899

[B21] Liu L. Y. Feng B. Chen J. Tan Q. R. Chen Z. X. Chen W. S. (2015). Herbal medicine for hospitalized patients with severe depressive episode: a retrospective controlled study. J. Affect. Disord. 170, 71–77. 10.1016/j.jad.2014.08.027 25233242

[B22] Liu X. Ouyang S. Yu B. Liu Y. Huang K. Gong J. (2010). PharmMapper server: a web server for potential drug target identification using pharmacophore mapping approach. Nucleic Acids Res. 38, W609–W614. 10.1093/nar/gkq300 20430828 PMC2896160

[B23] Liu X. Wei F. Liu H. Zhao S. Du G. Qin X. (2020). Integrating hippocampal metabonomics and network pharmacology deciphers the antidepressant mechanisms of Xiaoyaosan. J. Ethnopharmacol, 268, 113549. 10.1016/j.jep.2020.113549 33152435

[B24] Liu Y. Du T. Zhang W. Lu W. Peng Z. Huang S. (2019). Modified huang-lian-jie-du decoction ameliorates A. Oxid. Med. Cell Longev. 2019, 8340192. 10.1155/2019/8340192 31781354 PMC6875425

[B25] Liu Z. Guo F. Wang Y. Li C. Zhang X. Li H. (2016). BATMAN-TCM: a bioinformatics analysis tool for molecular mechANism of traditional Chinese medicine. Sci. Rep. 6, 21146. 10.1038/srep21146 26879404 PMC4754750

[B26] Manfredi R. Sabbatani S. (2010). Novel pharmaceutical molecules against emerging resistant gram-positive cocci. Braz. J. Infect. Dis. 14, 96–108. 10.1590/s1413-86702010000100020 20428664

[B27] Naziroglu M. Cig B. Ozgul C. (2014). Modulation of oxidative stress and Ca(2+) mobilization through TRPM2 channels in rat dorsal root ganglion neuron by *Hypericum perforatum* . Neuroscience 263, 27–35. 10.1016/j.neuroscience.2014.01.006 24434769

[B28] Okamoto H. Chino A. Hirasaki Y. Ueda K. Iyo M. Namiki T. (2013). Orengedoku-to augmentation in cases showing partial response to yokukan-san treatment: a case report and literature review of the evidence for use of these Kamp herbal formulae. Neuropsychiatric Dis. Treat. 9, 151–155. 10.2147/NDT.S38318 PMC3554226 23378767

[B29] Perez-Caballero L. Torres-Sanchez S. Romero-Lopez-Alberca C. Gonzalez-Saiz F. Mico J. A. Berrocoso E. (2019). Monoaminergic system and depression. Cell Tissue Res. 377, 107–113. 10.1007/s00441-018-2978-8 30627806

[B30] Reddy M. S. (2012). Depression - the global crisis. Indian J. Psychol. Med. 34, 201–203. 10.4103/0253-7176.106011 23436954 PMC3573568

[B31] Rosel P. Arranz B. Urretavizcaya M. Oros M. San L. Navarro M. A. (2004). Altered 5-HT2A and 5-HT4 postsynaptic receptors and their intracellular signalling systems IP3 and cAMP in brains from depressed violent suicide victims. Neuropsychobiology 49, 189–195. 10.1159/000077365 15118356

[B32] Ru J. Li P. Wang J. Zhou W. Li B. Huang C. (2014). TCMSP: a database of systems pharmacology for drug discovery from herbal medicines. J. Cheminf. 6, 13. 10.1186/1758-2946-6-13 PMC4001360 24735618

[B33] Rubio J. Caldas M. Davila S. Gasco M. Gonzales G. F. (2006). Effect of three different cultivars of Lepidium meyenii (Maca) on learning and depression in ovariectomized mice. BMC Compl. Alternative Med. 6, 23. 10.1186/1472-6882-6-23 PMC1534053 16796734

[B34] Sasaki K. Sudo T. Kurusu T. Kiuchi T. Yoshizaki F. (2000). [The mechanism of alteration of monoamine metabolism in brain regions in marble burying behavior-isolated housing mice and effect of oren-gedoku-to on this alteration]. Yakugaku Zasshi 120, 559–567. 10.1248/yakushi1947.120.6_559 10860488

[B35] Steru L. Chermat R. Thierry B. Simon P. (1985). The tail suspension test: a new method for screening antidepressants in mice. Psychopharmacology (Berlin) 85, 367–370. 10.1007/BF00428203 3923523

[B36] Strachan M. W. (2005). Insulin and cognitive function in humans: experimental data and therapeutic considerations. Biochem. Soc. Trans. 33, 1037–1040. 10.1042/BST20051037 16246040

[B50] Trott O. Olson A. J. (2010). Autodockvina: Improving the speed and accuracy of docking with a new scoring function, efficient optimization, and multithreading. J. Comput. Chem., 31, 455–461. 10.1002/jcc.21334 19499576 PMC3041641

[B37] Valentine G. W. Mason G. F. Gomez R. Fasula M. Watzl J. Pittman B. (2011). The antidepressant effect of ketamine is not associated with changes in occipital amino acid neurotransmitter content as measured by [(1)H]-MRS. Psychiatr. Res. 191, 122–127. 10.1016/j.pscychresns.2010.10.009 PMC3061550 21232924

[B38] Wang P. Dai L. Zhou W. Meng J. Zhang M. Wu Y. (2019). Intermodule coupling analysis of huang-lian-jie-du decoction on stroke. Front. Pharmacol. 10, 1288. 10.3389/fphar.2019.01288 31772561 PMC6848980

[B39] Wang P. R. Wang J. S. Zhang C. Song X. F. Tian N. Kong L. Y. (2013). Huang-Lian-Jie-Du-Decotion induced protective autophagy against the injury of cerebral ischemia/reperfusion via MAPK-mTOR signaling pathway. J. Ethnopharmacol. 149, 270–280. 10.1016/j.jep.2013.06.035 23811213

[B40] Wang T. Wu Z. Sun L. Li W. Liu G. Tang Y. (2018). A computational systems pharmacology approach to investigate molecular mechanisms of herbal formula tian-ma-gou-teng-yin for treatment of Alzheimer’s disease. Front. Pharmacol. 9, 668. 10.3389/fphar.2018.00668 29997503 PMC6028720

[B51] Wigner P. Czarny P. Galecki P. SuK P. Sliwinski T. (2018). Themolecular aspects of oxidative & nitrosative stress and the tryptophan catabolites pathway (TRYCATs) as potential causes of depression. Psychiatry Res., 262, 566–574. 10.1016/j.psychres.2017.09.045 28951145

[B41] Wu L. M. Hu M. H. Tong X. H. Han H. Shen N. Jin R. T. (2012). Chronic unpredictable stress decreases expression of brain-derived neurotrophic factor (BDNF) in mouse ovaries: relationship to oocytes developmental potential. PLoS One 7, e52331. 10.1371/journal.pone.0052331 23284991 PMC3527516

[B42] Wu R. Wang H. Lv X. Shen X. Ye G. (2020). Rapid action of mechanism investigation of Yixin Ningshen tablet in treating depression by combinatorial use of systems biology and bioinformatics tools. J. Ethnopharmacol. 257, 112827. 10.1016/j.jep.2020.112827 32276008

[B43] Yu D. Tao B. B. Yang Y. Y. Du L. S. Yang S. S. He X. J. (2015). The Ido inhibitor coptisine ameliorates cognitive impairment in a mouse model of Alzheimer's disease. J Alzheimers Dis. 43, 291–302. 10.3233/JAD-140414 25079795

[B44] Yue G. H. Zhuo S. Y. Xia M. Zhang Z. Gao Y. W. Luo Y. (2014). Effect of huanglian jiedu decoction on thoracic aorta gene expression in spontaneous hypertensive rats. Evid Based Complement Alternat Med. 2014, 565784. 10.1155/2014/565784 24744811 PMC3976878

[B45] Zeng M. F. Pan L. M. Zhu H. X. Zhang Q. C. Guo L. W. (2010). Comparative pharmacokinetics of baicalin in plasma after oral administration of Huang-Lian-Jie-Du-Tang or pure baicalin in MCAO and sham-operated rats. Fitoterapia 81, 490–496. 10.1016/j.fitote.2010.01.004 20093170

[B46] Zhang K. Pan X. Wang F. Ma J. Su G. Dong Y. (2016). Baicalin promotes hippocampal neurogenesis via SGK1- and FKBP5-mediated glucocorticoid receptor phosphorylation in a neuroendocrine mouse model of anxiety/depression. Sci. Rep. 6, 30951. 10.1038/srep30951 27502757 PMC4977505

[B47] Zhang R. Zhu X. Bai H. Ning K. (2019). Network pharmacology databases for traditional Chinese medicine: review and assessment. Front. Pharmacol. 10, 123. 10.3389/fphar.2019.00123 30846939 PMC6393382

[B48] Zhang Y. Cao Y. Wang L. (2020). The effects of a new, improved Chinese medicine, Gengnianchun formula granules, on hot flushes, depression, anxiety, and sleep in Chinese peri- and postmenopausal women: a randomized placebo-controlled trial. Menopause. 27, 899–905. 10.1097/GME.0000000000001558 32379216

[B49] Zhu Y. L. Li S. L. Zhu C. Y. Wang W. Zuo W. F. Qiu X. J. (2020). Antidepressant metabonomics study of Danzhi Xiaoyao powder on rat model of chronic unpredictable mild stress(CUMS). J. Ethnopharmacol. 112832. 10.1016/j.jep.2020.112832 32387465

